# Excess BAFF May Impact HIV-1-Specific Antibodies and May Promote Polyclonal Responses Including Those from First-Line Marginal Zone B-Cell Populations

**DOI:** 10.3390/cimb46010003

**Published:** 2023-12-19

**Authors:** Kim Doyon-Laliberté, Matheus Aranguren, Josiane Chagnon-Choquet, Laurie-Anne Batraville, Olina Dagher, Jonathan Richard, Matteo Paniconi, Jean-Pierre Routy, Cécile Tremblay, Marie-Claude Quintal, Nathalie Brassard, Daniel E. Kaufmann, Andrés Finzi, Johanne Poudrier, Michel Roger

**Affiliations:** 1 Centre de Recherche du Centre Hospitalier de l’Université de Montréal (CRCHUM), Montréal, QC H2X 0A9, Canada; kim.doyon-laliberte@umontreal.ca (K.D.-L.); matheus.naegele.aranguren@umontreal.ca (M.A.); josiane.chagnon-choquet@umontreal.ca (J.C.-C.); laurie-anne.batraville@umontreal.ca (L.-A.B.); olina.dagher@umontreal.ca (O.D.); jonathan.richard.1@umontreal.ca (J.R.); c.tremblay@umontreal.ca (C.T.); nathalie.brassard.chum@ssss.gouv.qc.ca (N.B.); daniel.kaufmann@umontreal.ca (D.E.K.); andres.finzi@umontreal.ca (A.F.); 2 Département de Microbiologie, Infectiologie et Immunologie de l‘Université de Montréal, Montréal, QC H3T 1J4, Canada; matteo.paniconi@umontreal.ca; 3 Department of Medicine, McGill University Health Centre, McGill University, Montréal, QC H4A 3J1, Canada; jean-pierre.routy@mcgill.ca; 4 Centre Hospitalier Ste-Justine de l’Université de Montréal, Montréal, QC H3T 1C5, Canada; marie-claude.quintal@umontreal.ca; 5 Département de Médecine de l‘Université de Montréal, Montréal, QC H3T 1J4, Canada

**Keywords:** BAFF, HIV-1, B-cells, marginal zone, antibodies

## Abstract

We have previously shown that blood levels of B-cell Activating Factor (BAFF) rise relatively to disease progression status in the context of HIV-1 infection. Excess BAFF was concomitant with hyperglobulinemia and the deregulation of blood B-cell populations, notably with increased frequencies of a population sharing characteristics of transitional immature and marginal zone (MZ) B-cells, which we defined as marginal zone precursor-like” (MZp). In HIV-uninfected individuals, MZp present a B-cell regulatory (Breg) profile and function, which are lost in classic-progressors. Moreover, RNASeq analyses of blood MZp from classic-progressors depict a hyperactive state and signs of exhaustion, as well as an interferon signature similar to that observed in autoimmune disorders such as Systemic Lupus Erythematosus (SLE) and Sjögren Syndrome (SS), in which excess BAFF and deregulated MZ populations have also been documented. Based on the above, we hypothesize that excess BAFF may preclude the generation of HIV-1-specific IgG responses and drive polyclonal responses, including those from MZ populations, endowed with polyreactivity/autoreactivity. As such, we show that the quantity of HIV-1-specific IgG varies with disease progression status. In vitro, excess BAFF promotes polyclonal IgM and IgG responses, including those from MZp. RNASeq analyses reveal that blood MZp from classic-progressors are prone to Ig production and preferentially make usage of IGHV genes associated with some HIV broadly neutralizing antibodies (bNAbs), but also with autoantibodies, and whose impact in the battle against HIV-1 has yet to be determined.

## 1. Introduction

At present, more than 39 million individuals are living with the human immunodeficiency virus (HIV) [1]. Inflammation in the context of HIV establishes early and persists beyond therapy. This is likely to threaten immune competence, including mechanisms involved in maintenance of peripheral tolerance, suggesting that highly sophisticated immune reactions, such as germinal centers (GC), which generate memory and long-lived plasma cells guaranteeing high-affinity antibodies, may be overcome by less stringent extra-follicular responses, often associated with autoimmunity [2]. In this view, it was demonstrated that excess B-cell Activating Factor (BAFF) expression promotes T-independent extra-follicular responses, which are associated with antibodies of lower affinity/longevity [3]. Furthermore, excess BAFF has been shown to influence GC B-cell selection processes in the context of SLE [4], suggesting that in the context of excess BAFF, the affinity and function of antibodies recognizing HIV may be affected. We have previously shown that BAFF levels are in excess in the blood of HIV-1 rapid- and classic-progressors from the Montreal Primary HIV Infection (PHI) cohort early after infection and despite 9–12 months of antiretroviral therapy. We identified similar features in viremic slow-progressors from the Montreal Long Term Non-Progressor (LTNP) cohort, albeit to a lesser extent [5]. Interestingly, these features were not found in aviremic LTNP, also termed elite controllers (EC). Excess BAFF was thus concomitant with disease progression status and corresponded with hyperglobulinemia, which is a trait of polyclonal B-cell activation. Excess BAFF was also concomitant with a dysregulated B-cell compartment. Notably, we found high frequencies of a population sharing characteristics of transitional immature (TI) and marginal zone (MZ) B-cells, harboring the phenotype CD19+IgM+CD1c+CD27+ CD21loCD10+, which we designated “marginal zone precursor-like” (MZp) B-cells. These observations are consistent with BAFF being a potent survival, differentiation and activation factor for B-cells, especially MZ B-cell populations, which highly rely on BAFF signals for their selection [6]. Furthermore, since BAFF promotes NOTCH2 expression [7], this molecule is pivotal for the commitment to the MZ B-cell fate [8]. We have reported similar findings for HIV-1-infected Beninese commercial sex workers (CSWs) [9], and Simian Immunodeficiency Virus (SIV)-infected macaques [9], suggesting that excess BAFF and increased frequencies of MZp are a shared trait of HIV-1 infection, not restored via therapy. Moreover, we have shown that people living with HIV (PLHIV) from the Canadian HIV and Aging Cohort Study (CHACS) possess an excess level of BAFF in their blood and concomitant deregulated MZp even after 15 years of therapy. Importantly, these dysregulations were associated with accelerated atherosclerosis in these individuals [10].

Importantly, we have shown that in HIV-uninfected individuals, MZp present potent B-cell regulatory (Breg) capacities. Strikingly, our recent findings demonstrate that this Breg potential is altered in HIV-1 classic-progressors and is not restored upon treatment [11]. Moreover, we found that excess BAFF directly alters the Breg profile of MZp in vitro and affects their Breg function.

These findings led us to think that in the context of excessive BAFF, as encountered in HIV-1 infection, MZp may be driven to Ab production at the expense of their Breg competences. As such, MZ B-cell populations are known to generate “first-line” Ab responses via their polyreactive BCR and numerous Pattern Recognition Receptors (PRR) such as C-type lectins and Toll-like Receptors (TLR) [12]. Although MZ B-cells are associated with the production of IgM Abs, they have been shown to class-switch their immunoglobulins (Igs) from IgM to IgG or IgA in a BAFF-dependent manner via transmembrane activator calcium modulator and cyclophilin ligand interactor (TACI) [12], one of the three known receptors for BAFF amongst BAFF-Receptor (BAFF-R) and B-cell maturation antigen (BCMA) [13]. MZ B-cell populations are known to express high levels of TACI [14], and we have recently reported that MZp from the blood of HIV-1 classic-progressors express increased levels of TACI when compared to HIV-uninfected controls [11].

Importantly, recent RNASeq analyses done by our team showed that when compared to that of HIV-uninfected controls, MZp B-cells from the blood of HIV-1 classic-progressors are in a hyperactive state and present signs of exhaustion as they express high levels of T-box transcription factor TBX21 (T-bet) and CD11c, which are markers associated with extra-follicular responses and production of Igs of poor affinity [11,15]. MZp B-cells from the blood of classic-progressors highly express both IL-21R and TLR7, which along with T-bet and CD11c, are characteristics shared by a heterogeneous population found to be expanded in conditions such as autoimmunity, chronic viral infections, antibody-mediated rejection in organ transplants [16,17,18], and in critically ill patients with SARS-CoV-2 [19]. In a previous study with HIV-transgenic (Tg) mice [20], excess BAFF was concomitant with an enlarged splenic marginal zone, altered GC reactions, increased extra-follicular responses and high anti-dsDNA Ab titers, similar to that reported for BAFF-Tg mice, which present a break in tolerance and autoimmunity [20,21]. 

Altogether, these observations suggest that in the context of excess BAFF, extra-follicular Ab responses, such as those from MZ B-cell populations, may be favored at the expense of efficient GC reactions, and nourishing polyreactive, possibly autoreactive, low-affinity Ab responses, whose pertinence in the battle against HIV-1 have yet to be determined. Here, we performed functional assays, multiplexed analyses, and RNA sequencing to start addressing this important question.

## 2. Materials and Methods

### 2.1. Ethics Statement 

Written informed consent was obtained from all study participants [the Montreal Primary HIV-1 Infection Cohort [5,22] and Canadian Cohort of HIV-Slow-Progressors [23,24,25], and this research adhered to the ethical guidelines of Centre de Recherche du Centre Hospitalier de l’Université de Montréal (CRCHUM) and was reviewed and approved by the CRCHUM institutional review board (ethics committee, approval number CE 16.164-CA). This research adhered to the standards indicated by the Declaration of Helsinki. All participants were adults and provided informed written consent prior to enrolment in accordance with Institutional Review Board approval (project reference SL05.028).

### 2.2. Specimens

Cryopreserved blood specimens from ART-naïve male HIV-1 rapid- and classic-progressors, selected at the early phase of infection (i.e., 5–8 months after HIV acquisition), are from the same donors as previously described, from the Montreal PHI cohort specimen bank [5]. The date of infection had been estimated based on clinical and laboratory results, using criteria established by the Acute HIV Infection and Early Disease Research Program (National Institute of Allergy and Infectious Diseases [NIAID], Bethesda, MD, USA). We have also selected cryopreserved blood samples from male viremic slow-progressors and aviremic EC, which are also from the same donors as previously described, from the Montreal LTNP cohort specimen bank [26]. Plasma viral loads and blood CD4+ T-cell counts had been determined as reported [27] and can be found along with clinical data in our previous publication [5]. Briefly, rapid-progressors had CD4 T-cell counts below 250 cells/mm^3^ within 2 years of infection, with mean counts over time being 254 ± 118 cells/mm^3^ and, a count at early phase of 428 ± 148 cells/mm^3^ and viremia of 127 ± 184 × 10^3^ copies/mL. Classic-progressors had CD4 T-cell counts that remained above 500 cells/mm^3^ for the 2-year follow-up, with mean counts over time being 432 ± 140 cells/mm^3^ and, a count at early phase of 685 ± 185 cells/mm3 and viremia of 80 ± 100 × 10^3^ copies/mL. Slow-progressors had been infected for 8 years or more with CD4 T-cell counts above 500 cells/mm^3^ and low (viremic 3.3 ± 1.5 × 10^3^ copies/mL) to undetectable (aviremic < 0.005) viral loads in the absence of antiretroviral therapy (ART). Cryopreserved blood samples from male HIV-negative volunteers were obtained from the Montreal PHI cohort, and used as HIV-uninfected controls. Briefly, peripheral blood mononuclear cells (PBMCs) were isolated on Ficoll gradients, re-suspended in cryopreservation medium containing 90% heat-inactivated fetal bovine serum (hi-FBS) (Wisent Inc., Montreal, QC, Canada) and 10% dimethyl sulfoxide (DMSO), and stored in liquid nitrogen until use. PBMCs from healthy HIV negative donors were also obtained via leukapheresis and used as effector cells for measuring ADCC responses. Plasmas were stored at −80 °C until use. None of the subjects had syphilis, hepatitis B, or hepatitis C. 

### 2.3. HIV-Envelope (Env) IgG Measurements Assay 

The detection of HIV-Env-specific IgG in the plasma of HIV-1 rapid-, classic- and viremic slow-progressors was evaluated through a cell-based ELISA assay that expresses fully cleaved mature HIV-1 Env glycoprotein trimers at the surface of Hos cells, as described [28,29,30]. Briefly, 2 × 10^4^ Hos cells were plated in 96-well plates and transfected the next day using a standard polyethylenimine (PEI) transfection technique with 0.15 μg of the pSVIII plasmid expressing a cytoplasmic tail-deleted HIV1_YU2_ envelope glycoprotein and 0.01 µg of a Tat expressing plasmid per well, as reported [28,29,30]. Since the majority of antibodies elicited in PLHIV target CD4-induced Env epitopes [31], 0.007 μg of pCDNA3.1 CD4 expressing plasmid was added to the transfection mix [28,30]. The murine leukemia virus (MLV) Env was used as negative control. After 48 h, the transfected cells were washed and then incubated with a 1:1000 dilution of plasmas from PLHIV or 1 μg/mL of the gp120 outer-domain-recognizing antibody 2G12 (NIH AIDS Reagent Program, Manassas, VA, USA). Env-specific IgGs were detected using a horseradish peroxidase (HRP)-conjugated anti-human IgG-specific secondary Ab (Pierce) with a TriStar LB 941 luminometer (Berthold Technologies, Bad Wildbad, Germany). The level of Env-specific IgGs was calculated by taking the relative luminescence unit (RLU) value for each condition subtracted by the RLU obtained with the MLV Env. The data obtained were then normalized with the signal obtained with the anti-Env 2G12 Abs.

### 2.4. ADCC Measurement

We investigated whether Ig in plasma could eliminate gp120-coated target cells through ADCC, as described [32]. Recombinant HIV-1_YU2_ gp120 proteins were produced and purified as previously described [32,33]. CEM.NKr target cells were coated with 50 ng of recombinant HIV-1 gp120 (per 7.5 × 10^5^ cells) for 30 min at 37 °C before being stained for 20 min with a viability marker (AquaVivid, Invitrogen by Thermo Fisher Canada, Burlington, ON, Canada) and a cellular marker (eFluor 670, eBiosciences by Thermo Fisher Canada). Cryopreserved PBMCs from HIV-1-uninfected controls were labeled with a cellular marker (eFluor450, eBiosciences) and used as effector cells in this assay. Cell labeling was performed according to the manufacturer’s directions. Target and effector cells (at a ratio of 10:1, respectively) were placed together and incubated for 4–6 h with plasmas diluted (1:1000) or 5 μg/mL of the anti-cluster A Abs A32. The cells were then fixed with 2% phosphate-buffered saline (PBS)-formaldehyde solution containing a constant number of flow-cytometry particles (5 × 10^4^/mL) (AccuCount Blank Particles, 5.3 lm; Spherotech, Lake Forest, IL, USA). A constant number of particles (1 × 10^3^) was counted during cytometry acquisition in order to normalize the number of viable targets cells that survived. The normalized number of viable target cells was used to calculate the percentage (%) of ADCC-mediated killing using the following formula: (normalized number of viable target cells in targets plus effector) − (normalized number of viable target cells in targets plus effector plus plasma or Abs)/(normalized number of viable target cells in targets). The % of ADCC response obtained with each plasma was normalized with the response obtained with the anti-Env A32 Abs. Each sample was acquired with an LSRII, and data analysis was performed using FlowJovX.0.6.23.

### 2.5. Cell Sorting of Human Blood MZ and MZp B-Cells, RNA Isolation and Sequencing

As previously described [11,26], PBMCs from 3 HIV-1-uninfected controls, 3 HIV-1 classic-progressors at 5–8 months post-infection, and 3 HIV-1 EC selected as described above were thawed, washed in IMDM (Iscove’s Modified Dulbecco’s Medium, Gibco Life Technologies, New York, NY, USA), and processed for cell sorting with a FACSAriaIII apparatus. Live/dead exclusion was performed using LIVE/DEAD Fixable Aqua Dead Cell Stain (Invitrogen Thermo Fisher Scientific, Eugene, OR, USA). Non-specific binding sites were blocked using fluorescence-activated cell sorting (FACS) buffer (1× PBS, 2% hi-FBS) supplemented with 20% hi-FBS and 10 μg mouse IgG (Sigma-Aldrich, St-Louis, MO, USA). The cells were stained using the following conjugated mouse anti-human monoclonal antibodies (mAbs): PacificBlue-anti-CD19, APC-Cy7-anti-CD10 (BioLegend, San Diego, CA, USA), AlexaFluor700-anti-CD27, FITC-anti-IgM, PE-anti-CD21 (BD-Biosciences, Mississauga, ON, Canada), PerCP-eFluor710-anti-CD1c (eBioscience). Sorted live CD19+CD1c+IgM+CD27+CD21hiCD10− mature MZ and CD19+CD1c+IgM+CD27+CD21loCD10+ MZp B-cells were >95% pure. Total RNA was extracted using RNeasy Micro Kit (Qiagen, Toronto, ON, Canada) according to the manufacturer’s instructions. RNA integrity was validated using an RNA Pico Chip on the Agilent BioAnalyzer 2100, and RNA was sent to the IRIC Genomics Core Facility for RNAseq transcriptomic profiling and analysis. Libraries were prepared using Clontech Ultra Low RNA SMARTer v4 (Takara Bio, San Jose, CA, USA) and sequenced on a HiSeq2000. Genes with false discovery rate (FDR) values < 0.05 were considered to be differentially expressed. Gene expression levels were compared using raw read counts and the negative binomial distribution model implemented in DESEq2 [34], a differential expression analysis package developed for R, which adjusts for sample variations with the assumption that the vast majority of genes should have correlating expression levels. More specifically, the regularized log transformation (rlog) implemented in DESeq2 was used to transform raw data into log2 (readcount) for analysis and visualization.

### 2.6. Gene Set Enrichment Analyses (GSEAs)

Gene Set Enrichment Analyses (GSEAs) were produced using the software application GSEA 4.1.0, developed by the Broad Institute, as previously published [35,36]. Required input files are the expression data, the phenotype labels, and the gene set. The expression data file consists of a table whose first column is the gene name, followed by the gene description, followed by the genes’ expression values for each sample. The phenotype labels file associates a phenotype label (e.g., HIV+ or HIV−) with each sample in the group. The gene set for this manuscript (REACTOME_ANTIGEN_ACTIVATES_B_CELL_RECEPTOR_BCR_LEADING_TO_GENERATION_OF_SECOND_MESSENGERS) was downloaded from the Broad Institute’s Molecular Signatures Database (MSigDB) v.7.4. it is the B-cell activation pathway leading to the production of antibodies. The software generates an analysis for each gene set containing the Net Enrichment Score (NES), false discovery ratio (q-value), and *p*-value, as well as a heat map illustrating the gene expression variances between the two groups. The gene set is considered significantly modulated if q-value and *p*-value < 0.05. 

### 2.7. Human Tonsillar B-Cells

Human tonsils from HIV-uninfected individuals, who had undergone surgical tonsillectomy, were mechanically processed and the cells were cryopreserved in liquid nitrogen until use, as described above. The cells were thawed, washed in IMDM and B-cells were negatively enriched > 95% via an immunomagnetic-based technology (Dynabeads Untouched Invitrogen). Total B-cells or MZp sorted as described above were subsequently cultured in round bottom 96-well or 384-well microplates, at a concentration of 10^6^ cells/mL in IMDM supplemented with 10^−4^ β-2-mercaptoethanol, 10% hi-FBS and 1% penicillin/streptomycin, in absence or presence of stimuli (human recombinant soluble BAFF/BLyS/TNFSF13B Protein, R&D Systems, at 50–500 ng/mL, and/or TLR7 agonist at 2.5 μg/mL (Imiquimod (R837), Invitrogen) for 7 days at 37 °C and 5% CO_2_. Supernatants were then recovered and frozen at −20 °C until used. 

### 2.8. Ig Isotypes Measurements in Culture Supernatants

Culture supernatants were assessed for Ig isotypes concentrations using Milliplex map human immunoglobulin isotyping magnetic bead panel (MilliporeSigma, Etobicoke, ON, Canada) according to the manufacturer’s protocol. Data acquisition was carried out using Luminex 200 System (Luminex Corporation, Austin, TX, USA).

### 2.9. Multicolor Flow-Cytometry and gp120 Binding

PBMCs and total tonsillar B-cells were processed for flow-cytometry as previously described [26]. Briefly, live/dead exclusion was performed using Aqua-LIVE/DEAD Fixable Stain (Invitrogen Life technologies, Eugene, OR, USA). Non-specific binding sites were blocked using FACS buffer (1× PBS, 2% hi-FBS, and 0.1% sodium azide) supplemented with 20% hi-FBS, 10 µg mouse IgG (Sigma-Aldrich, St-Louis, MO, USA) and 5 µg Human BD FCBlock (BD Biosciences). The following mouse anti-human conjugated mAbs were used to detect extracellular markers on B-cells: APC Anti-CD19, BB515 Anti-IgM, AlexaFluor700-anti-CD27, BV421 Anti-CD10 (BD Biosciences), PerCP-eFluor 710 Anti-CD1c (eBioscience, San Diego, CA, USA). We had two different staining cocktails, each identical except for a variation for the PE slot, i.e., (1) PE-anti-CD21 (BD Bioscience) to verify that the MZp were indeed CD21lo when compared to CD21hi MZ, and (2) to verify gp120 binding by using biotinylated fully glycosylated or mutated gp120 (Biotin rgp120 HIV-1 IIIB, ImmunoDx, Woburn, MA, USA) and subsequent PE-conjugated streptavidin (Streptavidin-PE, BD-Biosciences), in presence or absence of a pre-incubation with mannose (5 μg/mL for 40 min on ice), as previously described [9]. Data acquisition was performed with FACSFortessa (BD-Biosciences) for blood PBMC samples, and LSRIIB (BD-Biosciences) for tonsillar samples. Analyses were carried out with FlowJo 10 software and GraphPad Prism. All stainings were compared to that of fluorescence minus one (FMO) values and isotype controls. Anti-mouse Ig(κ) Compbeads and CS&T Beads were used to optimize fluorescence compensation settings and calibrate the LSRIIB, respectively.

### 2.10. Statistical Analyses

The statistical significance of the differences between groups was assessed with a one-way ANOVA with post hoc Tukey test for data normally distributed, or otherwise with Kruskal–Wallis test with post hoc Dunn test. Normality was assessed with the Shapiro–Wilk test. For the correlations, a Pearson correlation was used if data were found to be normally distributed; otherwise, Spearman correlation was used instead. Analyses were performed using GraphPad Prism 9.1.1, on Mac. The Wald Test with Benjamini–Hochberg correction was used for RNAseq analysis. The results were considered significant when *p* < 0.05.

## 3. Results

### 3.1. HIV-1 Env-Specific IgG Levels in the Blood of PLHIV Are Reflective of Disease Progression Status and Related Intensity of Excess BAFF

**Figure 1:**
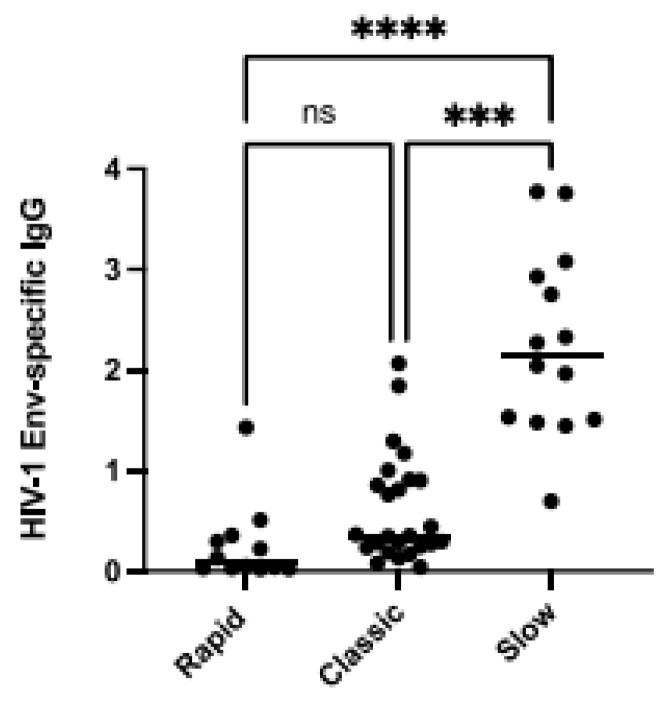
Elevated BAFF levels are associated with lower HIV-1-Envelope (Env)-specific IgG. HIV-1-Env-specific IgG levels in the plasma of HIV-1 rapid-, classic-, and slow-progressors in Relative Luminescence Units (RLU). Plasma IgG binding to cells co-expressing HIV-1YU2 Env∆CT was acquired in triplicates and normalized to signal values obtained for the gp120 outer domain-recognizing antibody 2G12. Statistical significance of differences between groups was assessed with a one-way ANOVA with post hoc Tukey test *** *p* < 0.001; **** *p* < 0.0001, non significant (ns).

We have previously reported that BAFF levels were in excess in the blood of PLHIV relative to their different disease progression statuses; rapid- (individuals whose HIV chronic phase is typically shorter than 2 years before progressing to AIDS), classic- (individuals whose HIV chronic phase typically lasts around 5 years before progressing to AIDS) and viremic slow-progressors (individuals whose HIV chronic phase is typically longer than 10 years before progressing to AIDS) from the Montreal PHI and LTNP cohorts. In this previous study, we had found that PHI rapid-progressors presented the highest levels of BAFF in their blood when compared to classic- and viremic slow-progressors [5], and classic-progressors presented greater levels than viremic slow-progressors, the latter in whom we had not found significantly elevated membrane forms when compared to HIV-1-uninfected controls [5]. Interestingly, excess BAFF was concomitant with hyperglobulinemia in these three groups of progressors, more so in rapid-progressors [5]. In order to verify whether this excessive BAFF context could impact the generation of HIV-1-specific antibodies, we have compared the HIV-1 Env-specific IgG levels in the blood of PLHIV from these three distinct groups of progressors. We show that viremic slow-progressors possess the highest concentrations of HIV-1 Env-specific IgG in their blood when compared to both rapid- (*p* = 0.0001) and classic-progressors (*p* = 0.001) (Figure 1). Classic-progressors did not present significantly greater HIV-1 Env-specific IgG levels than rapid-progressors. Of note, the correlations between BAFF levels and the HIV-1 Env-specific IgG levels in the blood of slow-progressors and ADCC activity against gp120-coated cells were not statistically significant. These data suggest that in these HIV-1 progressors, the quantity of HIV-1 Env-specific IgG may be reflective of disease progression status, and potentially affected by the extent of BAFF excess.

### 3.2. Influence of BAFF Levels on Ig Isotypes’ Production by Total Tonsillar B-Cells from Three Different HIV-Uninfected Donors 

**Figure 2:**
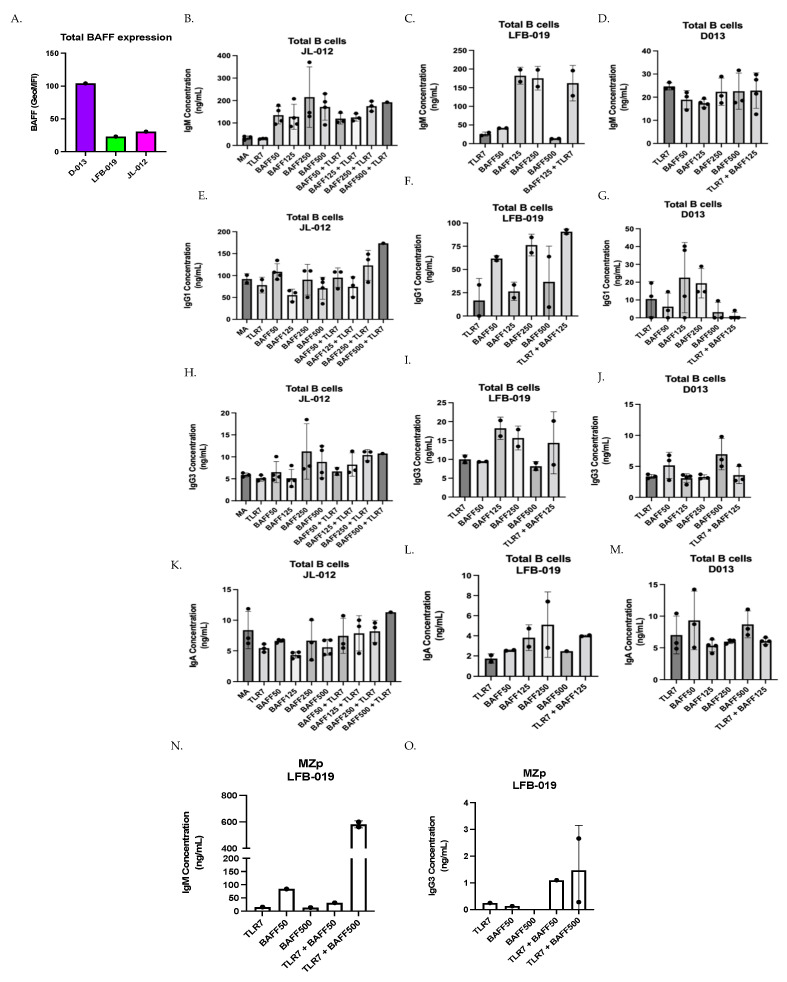
BAFF tends to modulate Ig production by total and MZp tonsillar B-cells. (**A**) Flow-cytometry analyses of ex vivo BAFF surface expression levels by total tonsillar cells from the tonsils of three different HIV-uninfected donors JL-012, LFB-019 and D013, as assessed via Geometric Mean of Fluorescence Intensity (GeoMFI). Multiplex analyses of concentrations of total IgM (**B**–**D**), IgG1 (**E**–**G**), IgG3 (**H**–**J**) and IgA (**K**–**M**) measured in day 7 supernatants of total tonsillar B-cells cultured either in medium alone (MA) or TLR7 agonist, with or without BAFF at 50–500 ng/mL. Data are presented for donors JL-012 (**B**,**E**,**H**,**K**) *n* = 3, LFB-019 (**C**,**F**,**I**,**L**) *n* = 2 and D013 (**D**,**G**,**J**,**M**) *n* = 3. Multiplex analyses of concentrations of total IgM (**N**), and IgG3 (**O**) in day 7 supernatants of sorted tonsillar MZp from donor LFB-013, cultured with TLR7 agonist, with or without BAFF at 50–500 ng/mL, *n* = 2. Statistical significance of differences between groups was assessed with Kruskal–Wallis test with post hoc Dunn test.

The above observations support the notion that in the context of excess BAFF, HIV-1-Env-specific IgG are quantitatively and qualitatively affected, possibly at the expense of non-specific Igs issued from polyclonal B-cell activation. While investigating the influence that different concentrations of soluble BAFF may exert on the production of total Ig isotypes by total tonsillar B-cells in vitro, we observed that the pattern of Ig isotypes produced in the presence of different soluble BAFF concentrations varied slightly between the three different donors (Figure 2B–M). This is reminiscent of our previous observations with tonsillar populations [11,26], and thus could possibly reflect an influence of tonsillar BAFF expression levels on shaping subsequent B-cell responsiveness to BAFF in vitro, since total tonsillar BAFF levels were different between each donor (Figure 2A). Upon analyzing Ig isotypes produced by total tonsillar B-cells, we were able to measure levels of IgM, IgG1, IgG3 and IgA but not those of IgG2 nor IgG4. We observed, for donor JL-012, that stimulation with BAFF alone enhanced production of IgM, more so at the highest concentrations of 250 and 500 ng/mL (Figure 2B). While IgG1 levels were not increased in the presence of BAFF alone, only slightly at 50 ng/mL (Figure 2E), we found that the production of IgG3 was increased in the presence of 250 and 500 ng/mL of BAFF (Figure 2H). Because of our interest in MZp, and the fact that blood MZp highly express TLR7 gene transcripts [26], which are increased in blood MZp from HIV-1 classic-progressors [11], we have chosen to co-stimulate with a TLR7 agonist. Culturing total tonsillar B-cells with TLR7 agonist alone was no different than that of medium alone (Figure 2B,E,H,K), whereas combining TLR7 agonist with BAFF at concentrations of 250 and 500 ng/mL tended to increase the production of all the measured isotypes in this donor (Figure 2B,E,H,K). 

Upon assessing Ig isotypes’ production by total tonsillar B-cells from donor LFB-019, which expressed the lowest levels of total tonsillar BAFF (Figure 2A), we also observed that BAFF alone can lead to the production of all the Ig isotypes measured, especially at concentrations of 125 and 250 ng/mL (Figure 2C,F,I,L), while at BAFF concentration of 500 ng/mL, Ig production was lower for all isotypes. Interestingly, combining TLR7 agonist with BAFF at 125 ng/mL increased IgG1 production over that of either stimulus used alone (Figure 2F).

When assessing Ig isotypes produced by total tonsillar B-cells from donor D013, we observed less Ig production, no matter the concentration of soluble BAFF added (Figure 2D,G,J,M), especially for IgM production (Figure 2D). A small IgG1 production can be observed at BAFF concentrations of 125 and 250 ng/mL (Figure 2G). Similarly, small IgG3 and IgA production can be observed at BAFF concentration of 500 ng/mL (Figure 2J,M). In this donor, which expresses the highest level of total tonsillar BAFF (Figure 2A), the TLR7 agonist when combined with BAFF did not impact on the Ig isotypes being produced. 

Again, because of our interest in MZp, and the fact that MZ B-cell populations have been shown to bind to HIV-Env, notably via TLR10 and C-type lectins [37,38], we have assessed the effect of adding gp120 in our total tonsillar B-cell cultures, as it was shown by other groups that this could activate and induce CSR of B-cells in vitro [37]. Binding to fully glycosylated gp120 is shown for both blood and tonsillar MZ and MZp and is reduced via mannose (Supplementary Figure S1). We find that BAFF slightly increased gp120 binding by MZ and MZp (Supplementary Figure S1) and that gene transcripts for C-type lectins such as CLEC2D are highly expressed or are increased in MZp from the blood of HIV-1 classic-progressors (Supplementary Figure S2). Interestingly, for total B-cell cultures from tonsillar donors LFB-013 and D013, adding gp120 Bal or IIIB, which have a tropism for CCR5 and CXCR4, respectively [39,40], seemed to lower the levels of IgM and IgG1 produced in response to TLR7 agonist combined with BAFF, but not those of IgG3 nor IgA (Supplementary Figure S3).

Although the above observations require further experimentation, as the small sample size making it difficult to make causational links, these results could suggest that the different expression levels of total tonsillar BAFF in our three different donors influence the concentrations at which soluble BAFF alone could exert an influence on Ig isotypes produced. For instance, when looking at IgG3, for donor JL-012, the effects of soluble BAFF tended to be optimum at concentrations of 250–500 ng/mL, whereas in donor LFB-013, which presented the lowest total tonsillar BAFF levels, the effects of soluble BAFF tended to be optimum at concentrations of 125–250 ng/mL. Interestingly, in donor D013, which presented the highest levels of total tonsillar BAFF, effects of soluble BAFF in vitro were rather observed at 500 ng/mL These results could suggest that BAFF levels in tonsillar tissues have an impact on the threshold for B-cell responsiveness to soluble BAFF in vitro, suggesting that donors with lower BAFF levels can better modulate their Ig responses when facing inflammatory signals. Moreover, our results could suggest that soluble BAFF at high concentrations ranging from 50–500 ng/mL promote non-specific polyclonal Ig production by total tonsillar B-cells.

### 3.3. Effect of BAFF Levels on Ig Production by Sorted Tonsillar MZp

We have previously shown that tonsillar MZp from donor LFB-019 presented efficient Breg function in vitro, which was significantly reduced upon the addition of 500 ng/mL of soluble BAFF [11]. This had led us to hypothesize that in conditions of excess BAFF, MZp may be driven to Ig production at the expense of their Breg capacities. In order to address this, sorted MZp from donor LFB-019 were cultured with TLR7 agonist and BAFF at concentrations of 50 and 500 ng/mL. We observe that IgM production was increased over that of either stimulus used alone, especially with soluble BAFF at 500 ng/mL (Figure 2N). Moreover, IgM concentrations in these cultures were comparable to those obtained with total tonsillar B-cells from this donor (Figure 2C). We could not detect IgG1, IgG2, IgG4 nor IgA production in these cultures, but could measure IgG3 in cultures with TLR7 agonist and BAFF (Figure 2O). Nevertheless, these results suggest that elevated BAFF levels can promote Ig production by tonsillar MZp B-cells, and that stimulation by a TLR7 agonist in presence of different BAFF concentrations can accentuate these responses. Altogether, the above observations support the notion that excess BAFF, such as encountered in HIV-1 progressors, could contribute to hyperglobulinemia reported for these individuals [37,41]. 

### 3.4. Blood MZp B-Cells from HIV Classic-Progressors Are More Prone to Ig Production When Compared to MZp from HIV-Uninfected Individuals 

**Figure 3:**
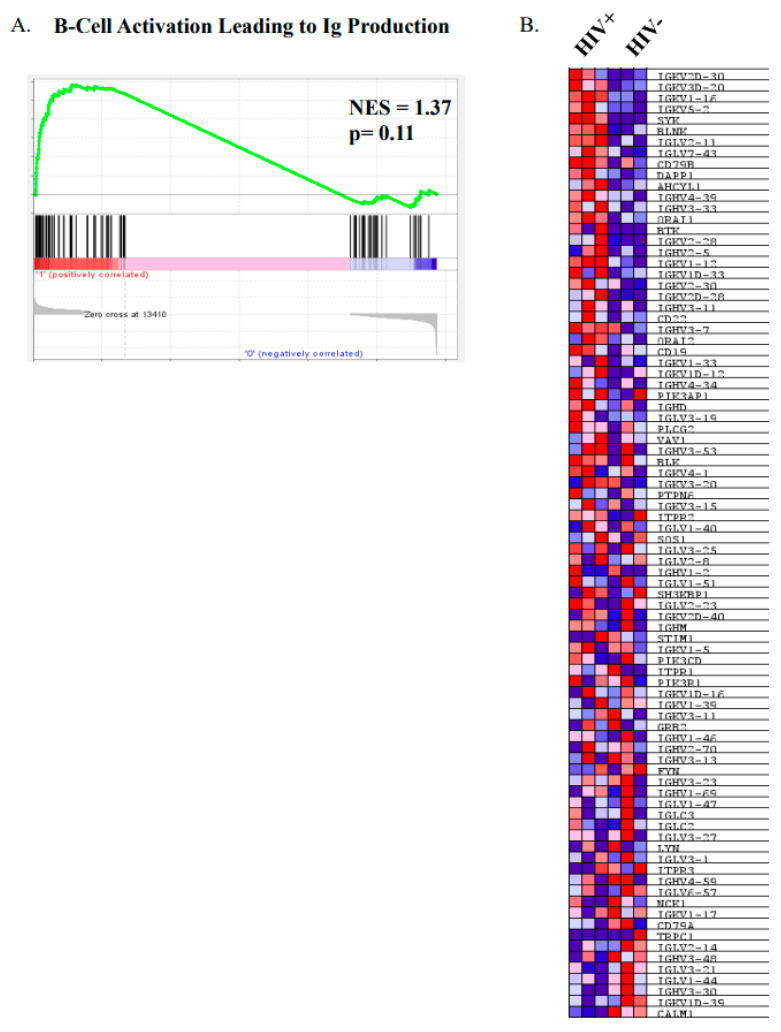
Precursor-like marginal zone (MZp) B-cells from the blood of HIV-1 classic-progressors possess an activated profile when compared to MZp from the blood of HIV-1-uninfected controls. Data exploration of the transcriptomic analyses via RNAseq of sorted mature marginal zone (MZ) and MZ precursor-like (MZp) B-cells from the blood of 5–8 months HIV-1 classic-progressors (HIV+), elite controllers (EC) and HIV-1-uninfected controls (HIV−), *n* = 3 of each group. (**A**) Gene Set Enrichment Analyses (GSEA) of “B-cell activation leading to immunoglobulin production” pathways in sorted blood MZp B-cells from HIV-1 classic-progressors when compared to HIV-1-uninfected controls. (**B**) Heatmap of transcripts of genes involved in activation leading to immunoglobulin production in blood MZp from HIV-1 classic-progressors and HIV-1-uninfected controls.

We have previously shown that MZp from HIV classic-progressors present an altered transcriptomic profile, with increased expression levels of IFN stimulated genes (ISG), TACI and exhaustion markers. Also, PI3K-AKT-mTOR and CREB/cJUN signatures were downregulated in MZp B-cells from the blood of these HIV-1 classic-progressors, which is overall suggestive of MZp being highly solicited and activated [11]. Thus, as depicted in Figure 3, we performed a GSEA, in which we used the Reactome database, a gene set that is related to Ig heavy and light chain usage. In Figure 3A, we can see that there is a trend for global upregulation of Ig gene usage for MZp from HIV+ vs. HIV−, suggesting that these cells might produce higher titers of antibodies with higher diversity when compared to their uninfected counterparts. In Figure 3B, we can see the same genes that were analyzed in Figure 3A in a heat map, allowing us to see which genes are upregulated or downregulated individually, in each group, suggesting that the activation pathway leading to Ig production by blood MZp from HIV-1 classic-progressors is not only different from that of HIV-1-uninfected controls, but also upregulated in the majority of the Ig transcripts evaluated in this analysis. These results suggest that blood MZp from HIV classic-progressors are more prone to Ig production when compared to MZp from HIV-uninfected individuals.

### 3.5. Blood MZp Present Transcripts for Ig Heavy Variable Chain (IGHV) Genes That Correlate with Usage by Broadly Neutralizing Antibodies and/or Autoantibodies

**Figure 4:**
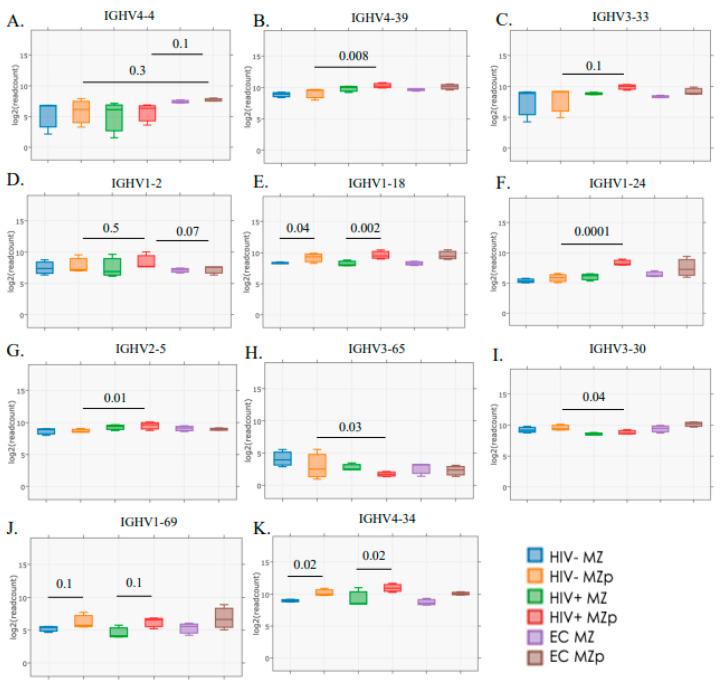
Transcriptomic analyses reveal that Ig variable heavy chain (IGVH) usage by blood precursor-like marginal zone (MZp) B-cells is associated with certain broadly neutralizing antibodies and/or autoimmunity and is modulated by the HIV context. RNAseq analyses of marginal zone (MZ) and precursor-like MZ (MZp) B-cells sorted from the blood of HIV-1-uninfected controls (HIV−), 5–8 months HIV-1 classic-progressors (HIV+) and elite controllers (EC), demonstrating gene expression levels of (**A**) IGHV4-4, (**B**) IGHV4-39, (**C**) IGHV3-33, (**D**) IGHV1-2, (**E**) IGHV1-18, (**F**) IGHV1-24, (**G**) IGHV2-5, (**H**) IGHV3-65, (**I**) IGHV3-30, (**J**) IGHV1-69 and (**K**) IGHV4-34. N = 3 for each study group. Statistical significance of differences between groups was assessed with the Wald Test with Benjamini–Hochberg correction.

Interestingly, upon analyzing IGHV gene usage, we found that blood MZp present higher transcripts for IGHV genes utilized in different groups of bNAbs. As such, for bNAbs recognizing the glycan dependent VI/V2/V3 region of gp120 [42], which are known for their autoreactive potential, it is possible to observe a higher tendency of IGHV4-4 gene usage for blood MZp from EC when compared to those of HIV classic-progressors (*p* = 0.1) or HIV-1-uninfected controls (*p* = 0.3) (Figure 4A). However, IGHV4-39 gene transcripts are significantly increased in blood MZp from HIV-1 classic-progressors when compared to HIV-uninfected controls (*p* = 0.008) (Figure 4B). A similar trend can be observed for IGHV3-33 gene expression (*p* = 0.1) (Figure 4C). Interestingly, some IGHV genes can be associated with bNAbs that recognize the CD4 binding site or the gp120/gp41 [42]. Some of them, such as IGHV1-2 (*p* = 0.5), IGHV1-18 (*p* = 0.3), IGHV1-24 (*p* = 0.0001) and IGHV2-5 (*p* = 0.01) present with a trend or significant increase in their gene transcripts in blood MZp from HIV-1 classic-progressors when compared to HIV-uninfected individuals (Figure 4D–G). As for IGHV1-18, gene transcripts are significantly higher in blood MZp from HIV classic-progressors when compared to more mature MZ from these same individuals (*p* = 0.002), or between MZp and mature MZ from HIV-1-uninfected individuals (*p* = 0.04). A downregulation of IGHV3-65 (*p* = 0.03) and IGHV3-30 (*p* = 0.04) gene transcripts in blood MZp from HIV classic-progressors when compared to HIV-uninfected controls could also be observed (Figure 4H,I). Interestingly, gene transcripts coding for IGHV utilized by Abs known to be autoreactive and increased in autoimmunity, such as SLE, multiple sclerosis (MS) or rheumatoid arthritis (RA) are increased in MZp when compared to MZ for each clinical group. A trend can be observed for IGHV1-69 gene transcripts between mature MZ and MZp from HIV classic-progressors (*p* = 0.1) or HIV-uninfected controls (*p* = 0.1). Those same observations are significantly different for IGHV4-34 (*p* = 0.02). Altogether, our results suggest that blood MZp from HIV classic-progressors have the potential to contribute to protective Abs but also the potential to produce autoreactive Abs, which could be harmful in the context of HIV-1. Further experimentation is required to verify whether these Abs possess any neutralizing activity.

## 4. Discussion

Our findings suggest that the quantity of HIV-1-Env-specific IgG may vary with the severity of disease progression status and possibly associated intensity of excess BAFF previously reported for these PLHIV [5]. These findings suggest that excess BAFF may influence IgG specificity and function, although this remains to be established at a larger scale. Although it may be argued that most individuals have never been exposed to HIV-1-YU2, and thus our method to detect Env-specific IgG may not be valid, we must point out that to measure the levels of HIV-1 Env-specific IgG, we used a cell-based ELISA assay in which HIV-1 YU2 Env was co-expressed with CD4 [30]. Upon CD4 interaction, the HIV-1 Env exposes conformational CD4-induced Env epitopes within the coreceptor binding site (CoRBS), the constant region 1 and 2 (C1-C2 or cluster A) and the gp41 immunodominant domain (gp41ID) [28,30,43]. These epitopes map highly conserved regions of the HIV-1 Env [28,43,44,45,46,47,48,49,50]. Antibodies targeting the CoRBS, the cluster A regions and the gp41ID are naturally elicited during HIV-1 and are consequently abundantly present in the vast majority of plasma of PLHIV [31,43,47,51,52,53,54]. Despite not been specifically exposed to HIV-1 YU2 Env, plasma from PLHIV therefore contains CD4-induced Abs able to recognize the CD4-bound YU2 Env.

Our results also suggest that excess BAFF may favor hyperglobulinemia issued from polyclonal B-cell activation, also reported for these individuals [5]. As such, we find that in tonsils from three different HIV-uninfected donors, high concentrations of soluble BAFF alone can modulate Ig isotypes’ production by total tonsillar B-cells, and that stimulation with a TLR7 agonist in the presence of different BAFF concentrations can accentuate Ig responses, including those of MZp. Our observations also point to a favoring of IgM and IgG3 isotypes being produced in response to soluble BAFF in our total and MZp tonsillar B-cell cultures, although the specificity of these Ig remains to be elucidated. We have found that total and MZp B-cells when cultured with 50 ng/mL soluble BAFF in vitro, downregulated BAFF-R and that MZ populations up-regulated TACI [11], which is in line with BAFF signaling being involved not only for survival but for Ig production and class switching. We found that Igs isotypes produced by tonsillar B-cells in vitro are likely the result of previously in situ acquired signals promoted by BAFF, and that of BAFF and/or TLR7 signals provided. Furthermore, we find that host total BAFF levels influence the threshold for total tonsillar B-cells responsiveness to soluble BAFF in vitro, and the outcome of Ig isotypes’ production, suggesting that BAFF levels in tonsillar tissue may modulate Ig responses from total tonsillar B-cells. As such, given that these B-cells came from tonsil samples that needed to be removed due to recurrent tonsillitis, it is expected that this tissue possess an elevated level of inflammation, a level that will, no doubt, vary depending on the donor. For instance, we think that B-cells from donor D-013 become accustomed to a high basal level of BAFF, so they need a much higher concentration of BAFF in order to respond to it. We have shown that depending on the donor, we need different levels of BAFF in order to induce a certain downregulation of NR4As and CD83 levels, and this seems to correlate with their BAFF base level [8]. Thus, when BAFF levels are higher in the environment, such as with tonsillar donor D013, B-cells may be affected in their overall capacity of Ig responses. Although the soluble BAFF concentrations we have used in vitro are much greater than that measured in the blood of HIV-1- progressors, they are consistent with concentrations used in similar studies [37], and we view they may reflect the extent of BAFF signals delivered in tissue.

Our observations with HIV-1 slow-progressors also suggest that the quantity of HIV-Env-specific IgG in their blood, herein in relation with their lower BAFF levels, may be linked to a certain degree of control in these individuals. Interestingly, we have shown that highly exposed seronegative (HESN) female commercial sex workers (CSWs) from Benin, which are a model of natural immunity against HIV, presented with lower levels of BAFF in their blood and vaginal tract when compared to HIV-infected CSWs and HIV-uninfected women from the general population, and this coincided with vaginal anti-gp41 IgG. Other groups have also shown the protective role of HIV-reactive antibodies in other HESN cohorts [55].

We show that the reduced quantity of HIV-1-Env-specific IgG measured in the blood of HIV-1 rapid- and classic-progressors is consistent with the perturbed lymphoid structures and altered GC capacities reported in the context of HIV-1 [20,56]. This is likely contributed to by several factors, such as reduced/altered T helper signals, altered follicular shuttling by MZ B-cell populations, persisting inflammation, etc. Accordingly, we reported lowered frequencies of memory B-cells in the blood of these HIV-1 progressors, whereas those of MZp were increased [5]. Similar to previous findings with HIV-Tg mice [20], where excess BAFF was concomitant with altered GC reactions and increased extra-follicular responses, our data also suggest that extra-follicular responses may be favored in HIV-1-progressors at the expense of efficient GC reactions, required for specific high-quality Abs. As such, blood MZp from HIV-1 classic-progressors presented increased gene transcripts for T-bet and CD11c [11], markers which are associated with extra-follicular B-cells. This is reminiscent of that observed in the context of moderate and severe SARS-CoV-2 infection [19], whereby elevated autoreactive Abs were associated with increased frequencies of B-cells presenting with a Tbet+CD11c+ extra-follicular profile and decreased GC reactions [19,57]. As such, we also previously observed autoimmunity in the HIV-Tg mouse system [20], and the hyperglobulinemia reported in our HIV-1 progressors is consistent with autoreactivity, as is the IGHV gene usage reported here for blood MZp.

The fact that the MZp Breg potential was found to be altered in HIV-1 progressors from the Montreal PHI cohort, and that host BAFF levels and excess BAFF can directly influence the Breg profile and function of tonsillar MZp in vitro [11], led us to hypothesize that in the context of excessive BAFF, such as that encountered in HIV-1 progressors, MZp may be driven to Ig production at the expense of their Breg competences. This is supported by our observations with sorted tonsillar MZp from donor LFB-013, shown to lose their Breg competence while producing IgM and IgG3 in presence of elevated BAFF concentrations [11]. Also, as mentioned earlier, BAFF-R gene transcripts levels were decreased in MZp from the blood of HIV-1 classic-progressors, whereas those of TACI were increased [11]. Furthermore, our GSEA shows that blood MZp from HIV classic-progressors are more prone to Ig production when compared to HIV-uninfected individuals. Recent RNASeq analyses of these MZp showed up-regulated BCMA and CD38 gene transcripts, suggestive of early blastic changes [11]. Also, the fact that we found increased α4β7 gene transcripts in our RNASeq analyses of these MZp could suggest their recruitment to mucosal sites, to possibly complete their differentiation scheme, as has been recently suggested [26]. In support of this, blood MZp from the same PHI HIV progressors presented with greater CCL20 and CCL25 migration indexes [26].

Our data suggest that in the context of HIV and persistent excessive BAFF environment, MZp may be highly solicited, selected, and expanded to contribute to hyperglobulinemia and the production of Abs, the pertinence of which remains to be established. As such, MZ B-cells have been shown to bind to the HIV-1 gp41, via TLR10 [37,38]. Moreover, MZ B-cells were also shown to bind to HIV-1 gp120 via C-type lectins [37,38], and BAFF increased gp120 binding and promoted the production of IgG and IgA, of which only a fraction recognized HIV [37]. This is consistent with our previous observations showing elevated expression levels of TLR10 gene transcripts [26], and gene transcripts for C-type lectins such as CLEC2D, which are highly expressed and/or increased by blood MZp from HIV-1 classic-progressors (Supplementary Figure S2). Moreover, blood MZp highly express gene transcripts for α4β7 integrins [26], shown to bind to HIV-Env [58]. We also find gp120 binding to MZ and MZp, and this is further increased by BAFF, and is reduced via mannose (Supplementary Figure S1). Interestingly, stimulation with gp120 seemed to lower the levels of IgM and IgG1 produced in response to TLR7 agonist combined with BAFF, but not those of IgG3 nor IgA (Supplementary Figure S3). Of interest, mice immunized with HIV-Env after treatment with BAFF presented increased MZ B-cells and produced neutralizing Ab responses [59], possibly either directly and/or via follicular shuttling and GC promotion [60]. Overall, these are suggestive of a certain level of contribution of MZ B-cell populations, such as MZp to the production of Abs in the context of HIV. 

Interestingly, transcriptomic analyses of blood MZp from HIV-1 classic-progressors show increased expression of IGHV gene transcripts associated with bNAbs and autoimmunity. It is important to highlight that this analysis was carried out solely on the light and heavy chain usages on a transcriptomic level. Abs undergo important changes between the gene transcription and their actual function. Nevertheless, given known evidence of autoreactivity in the HIV context and the fact that these chain usages are well known in other autoimmune contexts, we believe that this could suggest the potential of MZp to contribute to protective yet polyreactive and autoreactive Abs, which could possibly be harmful in the context of HIV.

IGHV1-24 gene transcripts were particularly increased in MZp from classic-progressors; this gene is used by the 21c Ab, known to be endowed with polyreactivity and autoreactivity, and its impact if abundantly produced may be of competing interest with perhaps more efficient Abs [61]. However, IGHV1-24 gene transcripts are also elevated in blood MZp from EC and may thus not be a total nuisance. Nevertheless, addressing these questions will require further investigation, and is outside the scope of this manuscript. 

MZp expressed higher levels of gene transcripts for IGHV4-34 and IGHV1-69, which are known for being highly expressed in autoimmune disorders such as SLE or in marginal zone lymphomas, in both PLHIV and non-infected individuals [42,62,63]. Our results also suggest that MZp possess a greater potential to produce autoreactive Abs than mature MZ. It is now known that CD27^+^IgG^+^ or CD27^+^IgA^+^ B-cells that express IGHV4-34 can recognize commensal bacteria [64]. It is possible that MZ populations are found within these populations since they are known for their responses against commensal bacteria in the mucosa [12]. The production of such polyreactive/autoreactive Abs could be beneficial in the short term to restrict the virus in the mucosa and stop its dissemination. However, whether Ab responses from MZ populations, especially in the context of excess BAFF, are beneficial in the long term remains to be elucidated since they could contribute to the autoimmune manifestations observed in PLHIV [61,65].

Further studies regarding existing strategies that block BAFF, already approved by the FDA for SLE treatment, could be explored to be administered as adjunct with existing antiretroviral therapy with a view to control excessive BAFF levels. This could possibly lower the inflammatory burden, ameliorate Breg function and promote efficient Ab responses against HIV-1. 

## 5. Conclusions

There are limitations in our study in terms of the methods and the number of samples used; as such, broader sampling will be mandatory in order to make strong conclusions. Also, without direct evidence of the mechanism through which BAFF affects the production of HIV-specific IgG, further research is needed to confirm our hypotheses. However, the present study highlighted the fact that excess BAFF may affect the efficiency of HIV-1-specific Ab responses and promote polyclonal B-cell responses, including those from MZp, likely at the expense of their Breg potential. The question as to whether MZp Ig’s contribution helps in the battle against HIV and should be more exploited, or if MZp contribute to hyperglobulinemia and should thus be eliminated remains unclear. 

## Data Availability

Data is contained within the article or Supplementary Material.

## Supplementary Materials

The following supporting information can be downloaded at: https://www.mdpi.com/article/10.3390/cimb46010003/s1, Figure S1: Marginal zone (MZ) and precursor-like (MZp) B-cell populations bind to fully glycosylated gp120 and this is increased by BAFF. Flow-cytometry analyses of negatively enriched total B-cells from the blood of an HIV-uninfected donor (A,B). Dot plots showing proportion of live gated blood CD19^+^ B-cells binding to fully glycosylated gp120 (top panels), and following pre-incubation of cells with mannose at 5 mg/mL for 40 min (bottom panels) (A). Gp120 binding B-cells where majorly IgM^+^ and comprised CD27^+^CD1c^+^CD21^+^ innate marginal zone (MZ) B-cells. Representative dot plots showing a negligible proportion of CD19^+^ B-cells binding to partially glycosylated gp120 (ΔV1-V2-V3) (B). Negatively enriched total B-cells from the tonsils of an HIV-uninfected donor (C–E). Dot plots showing proportion of live gated tonsillar CD19^+^ B-cells binding to fully glycosylated gp120 following culture with medium alone (MA) or BAFF at 125 ng/mL for 18 h (A). Frequencies of MZ (B) or MZp (C) binding to fully glycosylated gp120 following culture with MA or BAFF at 125 ng/mL. Figure S2: C-type lectins gene transcripts expressed by blood marginal zone (MZ) and precursor-like MZ (MZp) B-cells. RNAseq analyses of marginal zone (MZ) and precursor-like MZ (MZp) B-cells sorted from the blood of HIV-1-uninfected controls (HIV−), 5–8 months HIV-1 classic-progressors (HIV+) and elite controllers (EC), demonstrating gene expression levels of CLEC2A (A), DCIR (B), CLEC16A (C), α4 (D), β7 (E), DEC205 (F). N = 3 for each study group. * *p* < 0.05; ** *p* < 0.01; *** *p* < 0.001; **** *p* < 0.0001. The Wald Test with Benjamini–Hochberg correction was used for RNAseq analysis. Figure S3: Effect of adding gp120 on Ig production by total tonsillar B-cells. Multiplex analyses of concentrations of total IgM (A–E), IgG1 (B–F), IgG3 (C–G) and IgA (D–H) measured in day 7 supernatants of total tonsillar B-cells cultured either with medium alone (MA), TLR7 agonist or gp120 Bal or IIIB, with or without BAFF at 125 ng/mL. Data are presented for donors LFB-019 (A–D) *n* = 2 and D013 (E–H) *n* = 2.
