# Supplementary material for: Excess BAFF May Impact HIV-1-Specific Antibodies and May Promote Polyclonal Responses Including Those from First-Line Marginal Zone B-Cell Populations
Source: Curr Issues Mol Biol. 2023 Dec 19;46(1):25–43. doi: 10.3390/cimb46010003 (PMC10814910; doi:10.3390/cimb46010003)

Supplementary Figures

Figure Legends

Supplementary Figure S1. Marginal zone (MZ) and precursor-like (MZp) B-cell populations bind to fully glycosylated gp120 and this is increased by BAFF.

Flow cytometry analyses of negatively enriched total B-cells from the blood of an HIV-uninfected donor **(A-B)**. Dot plots showing proportion of live gated blood $CD19^{+}$ B-cells binding to fully glycosylated gp120 (top panels), and following pre-incubation of cells with mannose at 5 mg/ml for 40 minutes (bottom panels) **(A)**. Gp120 binding B-cells where majorly IgM^{+} and comprised $CD27^{+}CD1c^{+}CD21^{+}$ innate marginal zone (MZ) B-cells. Representative dot plots showing a negligible proportion of $CD19^{+}$ B-cells binding to partially glycosylated gp120 (ΔV1-V2-V3) **(B)**. Negatively enriched total B-cells from the tonsils of an HIV-uninfected donor **(C-E).** Dot plots showing proportion of live gated tonsillar $CD19^{+}$ B-cells binding to fully glycosylated gp120 following culture with medium alone (MA) or BAFF at 125 ng/mL for 18 hours **(A)**. Frequencies of MZ **(B)** or MZp **(C)** binding to fully glycosylated gp120 following culture with MA or BAFF at 125 ng/ml**.**

Supplementary Figure S2. C-type lectins gene transcripts expressed by blood marginal zone (MZ) and precursor-like MZ (MZp) B-cells.

RNAseq analyses of marginal zone (MZ) and precursor-like MZ (MZp) B-cells sorted from the blood of HIV-1-uninfected controls (HIV-), 5-8 months HIV-1-infected classic progressors (HIV+) and elite controllers (EC), demonstrating gene expression levels of CLEC2A **(A),** DCIR **(B)**, CLEC16A **(C)**, α4 **(D)**, β7 **(E),** DEC205 **(F)**. N=3 for each study group. *P < 0,05; ** P< 0,01; *** P< 0,001; ****P< 0,0001. The Wald Test with Benjamini-Hochberg correction was used for RNAseq analysis.

Supplementary Figure S3. Effect of adding gp120 on Ig production by total tonsillar B-cells.

Multiplex analyses of concentrations of total IgM **(A-E)**, IgG1 **(B-F)**, IgG3 **(C-G)** and IgA **(D-H)** measured in day 7 supernatants of total tonsillar B-cells cultured either with medium alone (MA), TLR7 agonist or gp120 Bal or IIIB, with or without BAFF at 125 ng/ml. Data are presented for donors LFB-019 **(A- D)** n=2 and D013 **(E- H)** n=2.

Supplementary Figure S1

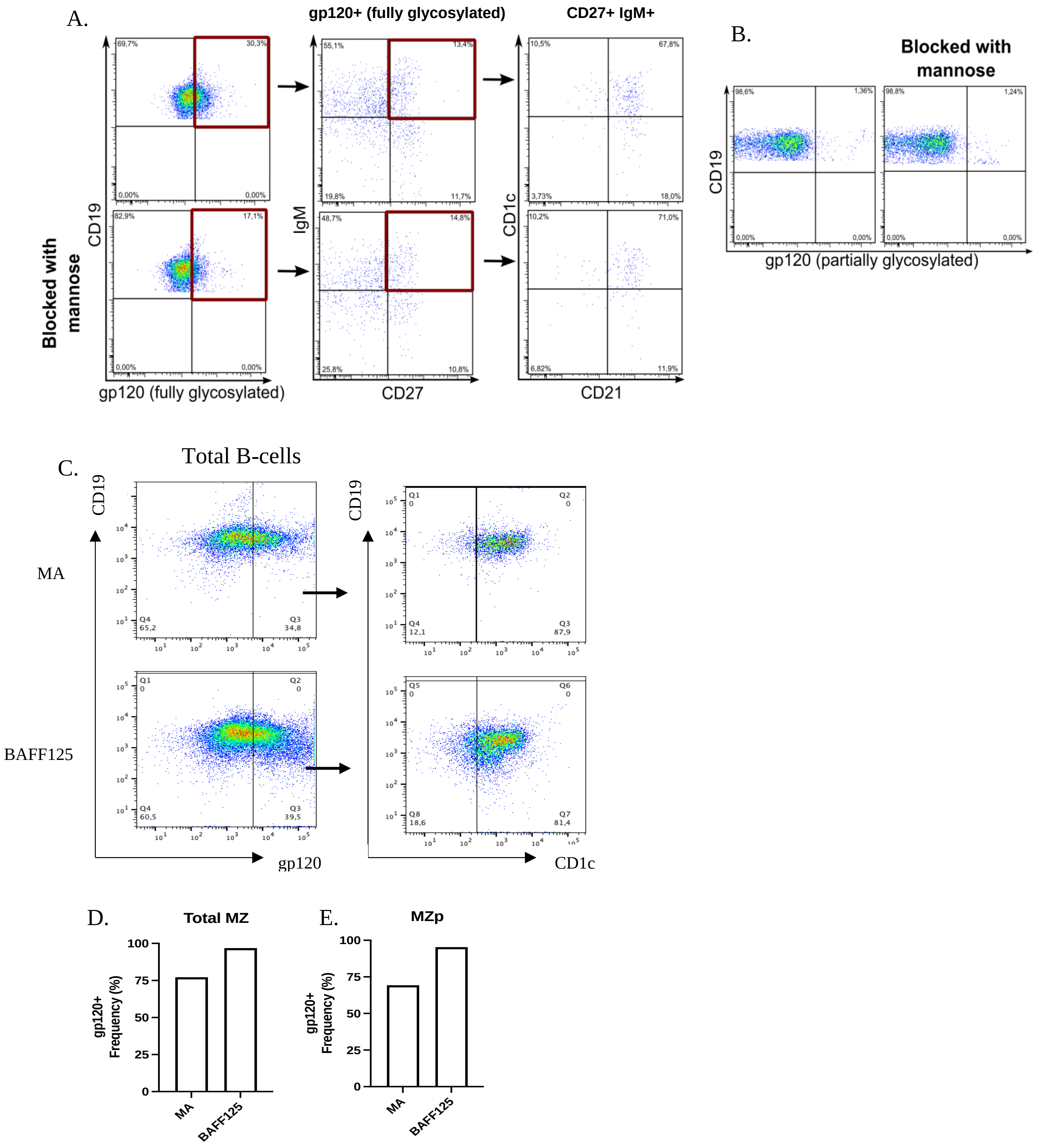

Supplementary Figure S2

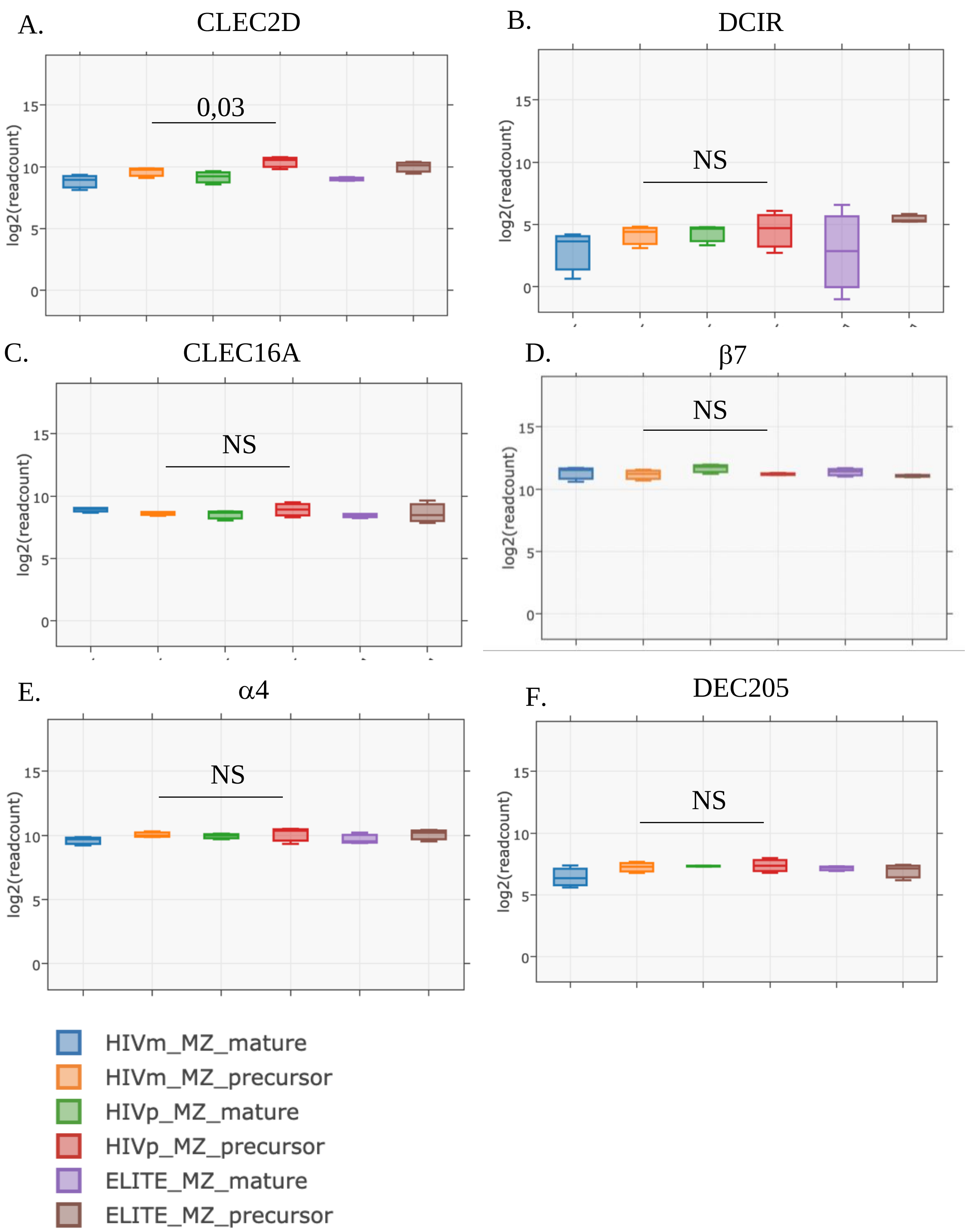

Supplementary Figure S3

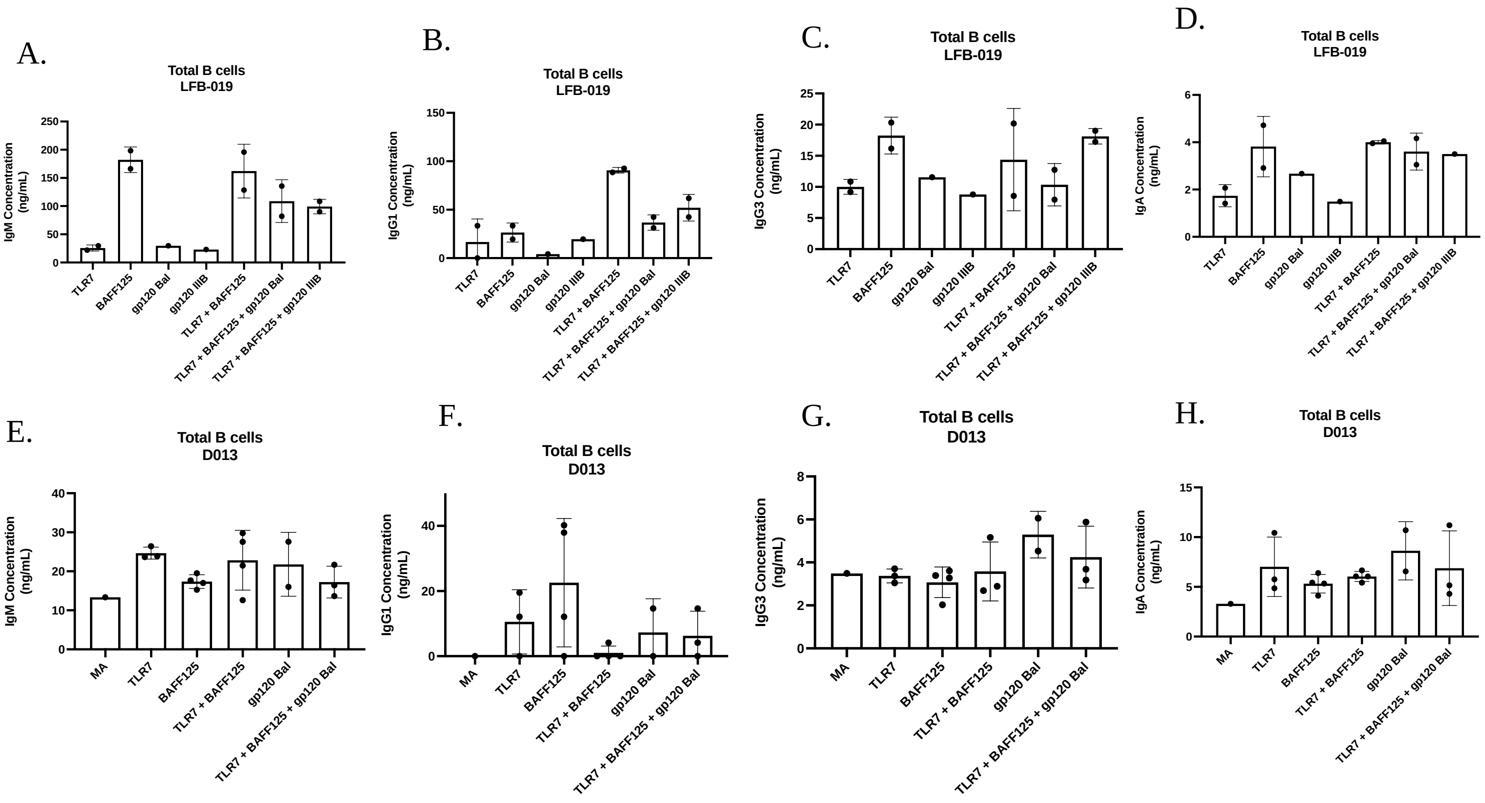

Supplement: Supplementary file 1 [file cimb-46-00003-s001.zip › cimb-2746826-supplementary.pdf]
